# Practical passive decoy state measurement-device-independent quantum key distribution with unstable sources

**DOI:** 10.1038/s41598-017-09367-y

**Published:** 2017-09-12

**Authors:** Li Liu, Fen-Zhuo Guo, Qiao-Yan Wen

**Affiliations:** 1 State Key Laboratory of Networking and Switching Technology, Beijing University of Posts and Telecommunications, Beijing, 100876 China; 2 School of Science, Beijing University of Posts and Telecommunications, Beijing, 100876 China

## Abstract

Measurement-device-independent quantum key distribution (MDI-QKD) with the active decoy state method can remove all detector loopholes, and resist the imperfections of sources. But it may lead to side channel attacks and break the security of QKD system. In this paper, we apply the passive decoy state method to the MDI-QKD based on polarization encoding mode. Not only all attacks on detectors can be removed, but also the side channel attacks on sources can be overcome. We get that the MDI-QKD with our passive decoy state method can have a performance comparable to the protocol with the active decoy state method. To fit for the demand of practical application, we discuss intensity fluctuation in the security analysis of MDI-QKD protocol using passive decoy state method, and derive the key generation rate for our protocol with intensity fluctuation. It shows that intensity fluctuation has an adverse effect on the key generation rate which is non-negligible, especially in the case of small data size of total transmitting signals and long distance transmission. We give specific simulations on the relationship between intensity fluctuation and the key generation rate. Furthermore, the statistical fluctuation due to the finite length of data is also taken into account.

## Introduction

Quantum key distribution (QKD) has been widely studied in both theoretical and experimental aspects^1–3^ since its initial proposal^4^. QKD enables two distant parties (Alice and Bob) to share a key, which is secret from any eavesdropper (Eve). It has been proved to be unconditional secure theoretically^5^.

Due to the imperfections of devices, there is still a big gap between the theory and practice of QKD. Fortunately, Lo *et al*. proposed a measurement-device-independent quantum key distribution (MDI-QKD) protocol^6^ to exclude all the attacks on detectors, which has been experimentally demonstrated by several groups^7–9^. Recently, the decoy state method has been widely used in MDI-QKD^9–17^ to defeat the photon number splitting (PNS) attack^18, 19^ and guarantee the security against imperfect sources, such as weak coherent pulses sources (WCPS)^20, 21^. These approaches are all related to the active decoy state selection, which is based on the assumption that Eve can not distinguish decoy and signal states. But this assumption may not stand in real active decoy state experiments, for which it may open up to side channels attacks and even break the security of the system when one actively modulates the intensities of pulses^22, 23^. The passive decoy state method^24–28^ can reduce the side channel information in the decoy state preparation procedure. Different from the active decoy state method, the passive one only uses one intensity signal, and Alice passively chooses the signal state and the decoy state according to the response of Alice’s detector. The method in ref. 28 extended passive decoy state to practical unstable light sources, which promoted its application to practical QKD. Therefore, it is necessary to consider the MDI-QKD with a passive decoy state. This has been demonstrated with phase encoding mode in ref. 29. Due to the different advantages between phase encoding and polarization encoding in practical application, we will apply the passive decoy state in MDI-QKD with polarization encoding mode^8, 9, 30, 31^.

An important imperfect factor of photon sources is intensity fluctuation^32^. Due to unavoidable interference from environments, there should be deviation between the true value and the assumed value. The deviation rises and falls irregularly, which can be called intensity fluctuation. The intensity fluctuation in experiments will result in the irregular change of the photon number distribution, and bring a potential security loopholes to the practical QKD^33^. The WCPs used in the passive decoy state method also has the imperfection of intensity fluctuation^34^. Therefore, how intensity fluctuations influence the performance of passive decoy state MDI-QKD protocol should also not be ignored.

In this paper, we apply the passive decoy state method to the MDI-QKD protocol with polarization encoding mode. Alice and Bob use WCPs with random phases to passively generate signal states or decoy states. Not only all the attacks on detectors can be removed, but also the side channels attacks on sources can be avoided, which may be generated by active modulation of source intensities. We analyse the security of this protocol, and show that MDI-QKD protocol with our passive decoy state method can provide a performance comparable to the active decoy state method. In order to fit for the demand of practical application, we discuss intensity fluctuation for MDI-QKD using the passive decoy state method. And based on the the formulas of yield and error rate derived in our paper, we get the key generation rate for our protocol with intensity fluctuation. According to the total gain and the overall error rate derived in our paper, we give a numerical simulations for our result. It shows that intensity fluctuation has a non-negligible effect on the key rate of the passive decoy state MDI-QKD protocol, especially in the case of small data size of total transmitting signals and long distance transmission. We give specific simulations on the relationship between intensity fluctuation and the key generation rate. Moreover, the finite-size analysis of this protocol is also taken into account in our paper.

## Results

### Passive Decoy State MDI-QKD Model

**Figure 1:**
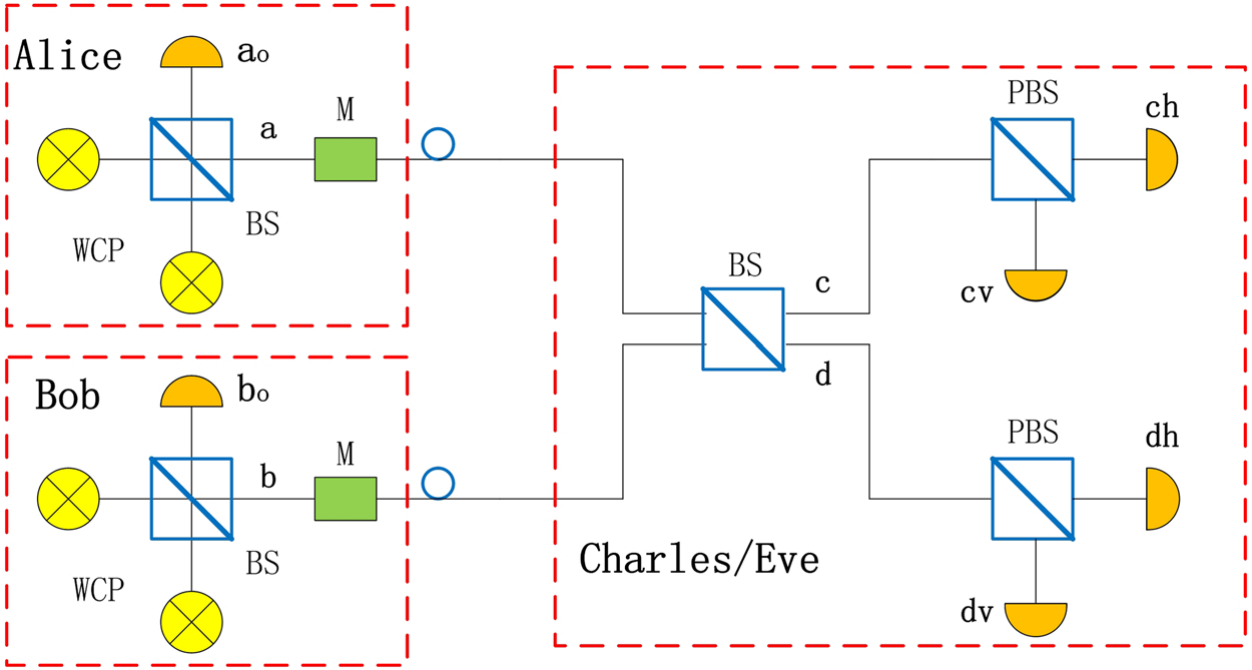
Passive decoy state MDI-QKD system model. WCP, weak coherent pulse; M, polarization modulators; BS, beam splitter; PBS, polarization BS; *a*_0_, *b*_0_, *c*_*h*_, *c*_*v*_, *d*_*h*_ and *d*_*v*_, photon detector.

In this section, we apply the passive decoy state method to the MDI-QKD protocol, as shown in Fig. 1. The general process of this protocol is described as follows.

Alice generates phase-randomized pulses using two weak coherent sources with intensities *μ*_1_ and *μ*_2_, respectively. These two pulses interfere at a beam splitter (BS) with a transmittance of 50%; then there are two outcome signals which have the classically correlated photon number statistics. Alice passively generates signal or decoy states. The state Alice generated is a joint-distribution state according to the result of detector *a*_0_. The detector *a*_0_ with two modes *c*_0_ and *c*_1_. The letter *c*_0_ indicates that the detector has no click and *c*_1_ indicates the detector has a click. Thus corresponding to the detector’s modes, the output *a* has two modes, *c*_0_ and *c*_1_, which describe the signal state and decoy state, respectively. The total probability of having *n* photons in the output light can be written as

1 $${p}_{n,a}^{t}=\frac{{\mu }^{n}}{n!}\frac{1}{2\pi }{\int }_{0}^{2\pi }\,{\alpha }^{n}{e}^{-\mu \alpha }d{\theta }_{a},$$

which is proven to be a non-Poissonian probability distribution^33^, and the parameters *μ* = *μ*_1_ + *μ*_2_, $\alpha =\tfrac{\tfrac{\mu }{2}+{\xi }_{a}\,\cos \,{\theta }_{a}}{\mu }$, ${\xi }_{a}=\sqrt{{\mu }_{1}{\mu }_{2}}$ and ${\theta }_{a}={\varphi }_{{a}_{2}}-{\varphi }_{{a}_{1}}$ is the phase difference. The joint probability of having *n* photons in mode *a* and no click in the detector *a*_0_ can be expressed by

2 $${p}_{n,a}^{{c}_{0}}=\mathrm{(1}-\varepsilon )\frac{{\mu }^{n}}{n!}{e}^{-{\eta }_{d}\mu }\frac{1}{2\pi }{\int }_{0}^{2\pi }\,{\alpha }^{n}{e}^{-\mathrm{(1}-{\eta }_{d})\mu \alpha }d{\theta }_{a},$$

$\epsilon$ is the dark count rate of detector, and *η*_*d*_ is the detector efficiency. The joint probability of having *n* photons in mode *a* and producing a click in the detector *a*_0_ has now the form

3 $${p}_{n,a}^{{c}_{1}}={p}_{n,a}^{t}-{p}_{n,a}^{{c}_{0}}.$$

Considering the normalization, the distributions of signal states and decoy states are respectively given by

4 $${q}_{n,a}^{{c}_{0}}={p}_{n,a}^{{c}_{0}}/({N}_{a}),\quad {q}_{n,a}^{{c}_{1}}={p}_{n,a}^{{c}_{1}}/\mathrm{(1}-{N}_{a}),$$

where

5 $${N}_{a}=\mathrm{(1}-\epsilon )\,{e}^{-{\eta }_{d}\mu }\frac{1}{2\pi }{\int }_{0}^{2\pi }\,{e}^{{\eta }_{d}\mu \alpha }d{\theta }_{a},$$

is a normalization constant.

Bob performs the same process as Alice. He generates phase-randomized pulses using two weak coherent sources with intensities *υ*_1_ and *υ*_2_, respectively. The distributions expressions of signal state ${p}_{m,b}^{{c}_{0}}$ and decoy state ${p}_{m,b}^{{c}_{1}}$ are just like those in Eq. (3). It can be given by

6 $$\begin{array}{rcl}{p}_{m,b}^{t} & = & \frac{{\upsilon }^{n}}{n!}\frac{1}{2\pi }{\int }_{0}^{2\pi }\,{\beta }^{n}{e}^{-\upsilon \beta }d{\theta }_{b},\\ {p}_{m,b}^{{c}_{0}} & = & \mathrm{(1}-\epsilon )\frac{{\upsilon }^{n}}{n!}{e}^{-{\eta }_{d}\upsilon }\frac{1}{2\pi }{\int }_{0}^{2\pi }\,{\beta }^{n}{e}^{-\mathrm{(1}-{\eta }_{d})\upsilon \beta }d{\theta }_{b},\\ {p}_{m,b}^{{c}_{1}} & = & {p}_{m,b}^{t}-{p}_{m,b}^{{c}_{0}},\end{array}$$

where the parameters are corresponding to Alice’s.

The main step of MDI-QKD based on BB84 protocol and here we adopt the polarization encoding method^6^. Each of Alice and Bob prepares phase-randomized WCP in a different BB84 polarization state which is selected by means of a polarization modulator (M), independently and at random for each signal. Then they send them to an untrusted relay Charles (or Eve), who is supposed to perform a Bell-state measurement(BSM). Inside the measurement device, signals from Alice and Bob interfere at a 50:50 beam splitter (BS) that has a polarizing beam splitter (PBS) on each end. The PBS projecting the input photons into either horizontal (H) or vertical (V) polarization states. A successful Bell state measurement corresponds to the observation of precisely two detectors (associated to orthogonal polarizations) being triggered. Charles announces the results through a public channel to Alice and Bob. According to the result that Charles announces, Alice and Bob proceed on to basis reconciliation, error correction, and privacy amplification, as in traditional QKD protocols^35^. Then both Alice and Bob can ensure they have the same bits.

### Estimation of the key generation rate

We modify the Gottesman-Lo-Lutkenhaus-Preskill (GLLP) formula^36^ according to the MDI-QKD security analysis. Then, we get the key generation rate formula,

7 $$R\geqslant {P}_{11}^{Z}{Y}_{11}^{Z}[1-H({e}_{11}^{X})]-{Q}_{{c}_{0}{c}_{0}}^{Z}{f}_{e}({E}_{{c}_{0}{c}_{0}}^{Z})\,H({E}_{{c}_{0}{c}_{0}}^{Z}),$$

where ${Y}_{11}^{Z}$ and ${e}_{11}^{X}$ are, respectively, the yield (the conditional probability that Charles declares a successful event) in the rectilinear (*Z*) basis and the error rate in the diagonal (*X*) basis, given that both Alice and Bob send single photon states; ${P}_{11}^{Z}$ denotes the probability distribution that both Alice and Bob send single photon states in the *Z* basis; *f*_*e*_ ≥ 1 is the efficiency of the error correction protocol; *H*(*x*) = −*x* *log*_2_ (*x*) − (1 − *x*) *log*_2_ (1 − *x*)) is the binary Shannon entropy function; ${Q}_{{c}_{0}{c}_{0}}^{Z}$ and ${E}_{{c}_{0}{c}_{0}}^{Z}$ denote, respectively, the total gain and quantum bit error rate (QBER) of signal state in the *Z* basis. Here we use the *Z* basis for key generation and the *X* basis for testing only.

In a MDI-QKD implementation with the model described in our paper, we can obtain the total gains and error rates in both the *X* basis and the *Z* basis,

8 $${Q}_{{c}_{i}{c}_{j}}^{\lambda }=\sum _{n,m=0}^{\infty }\,{q}_{n,a}^{{c}_{i}}{q}_{m,b}^{{c}_{j}}{Y}_{nm}^{\lambda },\quad {Q}_{{c}_{i}{c}_{j}}^{\lambda }{E}_{{c}_{i}{c}_{j}}^{\lambda }=\sum _{n,m=0}^{\infty }\,{q}_{n,a}^{{c}_{i}}{q}_{m,b}^{{c}_{j}}{Y}_{nm}^{\lambda }{e}_{nm}^{\lambda },$$

where *λ* ∈ {*X*, *Z*} denotes the basis choice and *i*, *j* = 0 or 1. ${Y}_{nm}^{\lambda }$ and ${e}_{nm}^{\lambda }$ are, respectively, the yield and error rate that Alice sends *n* photons pulse and Bob sends *m* photons pulse in the *λ* basis.

In practice, ${Q}_{{c}_{0}{c}_{0}}^{Z}$ and ${E}_{{c}_{0}{c}_{0}}^{Z}$ can be directly measured in experiments, while Alice and Bob only need to estimate the lower bound of the yield ${Y}_{11}^{Z}$ and the upper bound of the error rate ${e}_{11}^{X}$ using the decoy state methods. According to ref. 29, the lower bound of ${Y}_{11}^{\lambda }$ can be given

9 $$\begin{array}{rcl}{Y}_{11}^{\lambda }\geqslant \underline{{Y}_{11}^{\lambda }} & = & ({c}_{{c}_{1}{c}_{1}}{Q}_{{c}_{1}{c}_{1}}^{\lambda }+{c}_{{c}_{1}{c}_{0}}{Q}_{{c}_{1}{c}_{0}}^{\lambda }+{c}_{{c}_{0}{c}_{1}}{Q}_{{c}_{0}{c}_{1}}^{\lambda }+{c}_{{c}_{0}{c}_{0}}{Q}_{{c}_{0}{c}_{0}}^{\lambda }-{c}_{{c}_{1}0}{Q}_{{c}_{1}0}^{\lambda }\\  &  & -{c}_{{c}_{0}0}{Q}_{{c}_{0}0}^{\lambda }-{c}_{0{c}_{1}}{Q}_{0{c}_{1}}^{\lambda }-{c}_{0{c}_{0}}{Q}_{0{c}_{0}}^{\lambda }+{c}_{00}{Q}_{00}^{\lambda })\\  &  & \times {[(K-{A}_{1}{B}_{1})({q}_{\mathrm{0,}a}^{{c}_{0}}{q}_{\mathrm{1,}a}^{{c}_{0}}+{q}_{\mathrm{0,}a}^{{c}_{1}}{q}_{\mathrm{1,}a}^{{c}_{1}})({q}_{\mathrm{0,}b}^{{c}_{0}}{q}_{\mathrm{1,}b}^{{c}_{0}}+{q}_{\mathrm{0,}b}^{{c}_{1}}{q}_{\mathrm{1,}b}^{{c}_{1}})]}^{-1},\end{array}$$

where *λ* = *X* or *Z* and the coefficients of the total gain in each mode are

10 $$\begin{array}{ll}{c}_{{c}_{1}{c}_{1}}\,=\,K{q}_{\mathrm{0,}a}^{{c}_{1}}{q}_{\mathrm{0,}b}^{{c}_{1}}-{q}_{\mathrm{0,}a}^{{c}_{0}}{q}_{\mathrm{0,}b}^{{c}_{0}}, & {c}_{{c}_{1}{c}_{0}}\,=\,K{q}_{\mathrm{0,}a}^{{c}_{1}}{q}_{\mathrm{0,}b}^{{c}_{0}}+{q}_{\mathrm{0,}a}^{{c}_{0}}{q}_{\mathrm{0,}b}^{{c}_{1}},\\ {c}_{{c}_{0}{c}_{1}}\,=\,K{q}_{\mathrm{0,}a}^{{c}_{0}}{q}_{\mathrm{0,}b}^{{c}_{1}}+{q}_{\mathrm{0,}a}^{{c}_{1}}{q}_{\mathrm{0,}b}^{{c}_{0}}, & {c}_{{c}_{0}{c}_{0}}\,=\,K{q}_{\mathrm{0,}a}^{{c}_{0}}{q}_{\mathrm{0,}b}^{{c}_{0}}-{q}_{\mathrm{0,}a}^{{c}_{1}}{q}_{\mathrm{0,}b}^{{c}_{1}},\\ \,{c}_{{c}_{1}0}\,=K{q}_{\mathrm{0,}a}^{{c}_{1}}[({q}_{\mathrm{0,}b}^{{c}_{1}}{)}^{2}+{({q}_{\mathrm{0,}b}^{{c}_{0}})}^{2}], & \,{c}_{{c}_{0}0}=\,K{q}_{\mathrm{0,}a}^{{c}_{0}}[({q}_{\mathrm{0,}b}^{{c}_{1}}{)}^{2}+{({q}_{\mathrm{0,}b}^{{c}_{0}})}^{2}],\\ \,{c}_{0{c}_{1}}\,=\,K{q}_{\mathrm{0,}b}^{{c}_{1}}[({q}_{\mathrm{0,}a}^{{c}_{1}}{)}^{2}+{({q}_{\mathrm{0,}a}^{{c}_{0}})}^{2}], & \,{c}_{0{c}_{0}}=K{q}_{\mathrm{0,}b}^{{c}_{0}}[({q}_{\mathrm{0,}a}^{{c}_{1}}{)}^{2}+{({q}_{\mathrm{0,}a}^{{c}_{0}})}^{2}],\\ \,{c}_{00}\,=\,K[({q}_{\mathrm{0,}a}^{{c}_{1}}{)}^{2}+{({q}_{\mathrm{0,}a}^{{c}_{0}})}^{2}]\,[({q}_{\mathrm{0,}b}^{{c}_{1}}{)}^{2}+{({q}_{\mathrm{0,}b}^{{c}_{0}})}^{2}], & \\ \quad K\,=\,min\{{A}_{1}{B}_{2},{A}_{2}{B}_{1},{A}_{2}{B}_{2}\} & \end{array}$$

and

11 $$\begin{array}{c}{A}_{1}\,=\,\frac{{q}_{\mathrm{0,}a}^{{c}_{1}}{q}_{\mathrm{1,}a}^{{c}_{0}}-{q}_{\mathrm{0,}a}^{{c}_{0}}{q}_{\mathrm{1,}a}^{{c}_{1}}}{{q}_{\mathrm{0,}a}^{{c}_{0}}{q}_{\mathrm{1,}a}^{{c}_{0}}+{q}_{\mathrm{0,}a}^{{c}_{1}}{q}_{\mathrm{1,}a}^{{c}_{1}}},{A}_{2}\,=\,\frac{{q}_{\mathrm{0,}a}^{{c}_{1}}{q}_{\mathrm{2,}a}^{{c}_{0}}-{q}_{\mathrm{0,}a}^{{c}_{0}}{q}_{\mathrm{2,}a}^{{c}_{1}}}{{q}_{\mathrm{0,}a}^{{c}_{0}}{q}_{\mathrm{2,}a}^{{c}_{0}}+{q}_{\mathrm{0,}a}^{{c}_{1}}{q}_{\mathrm{2,}a}^{{c}_{1}}},\\ {B}_{1}\,\,=\,\frac{{q}_{\mathrm{0,}b}^{{c}_{1}}{q}_{\mathrm{1,}b}^{{c}_{0}}-{q}_{\mathrm{0,}b}^{{c}_{0}}{q}_{\mathrm{1,}b}^{{c}_{1}}}{{q}_{\mathrm{0,}b}^{{c}_{0}}{q}_{\mathrm{1,}b}^{{c}_{0}}+{q}_{\mathrm{0,}b}^{{c}_{1}}{q}_{\mathrm{1,}b}^{{c}_{1}}},{B}_{2}\,\,=\,\frac{{q}_{\mathrm{0,}b}^{{c}_{1}}{q}_{\mathrm{2,}b}^{{c}_{0}}-{q}_{\mathrm{0,}b}^{{c}_{0}}{q}_{\mathrm{2,}b}^{{c}_{1}}}{{q}_{\mathrm{0,}b}^{{c}_{0}}{q}_{\mathrm{2,}b}^{{c}_{0}}+{q}_{\mathrm{0,}b}^{{c}_{1}}{q}_{\mathrm{2,}b}^{{c}_{1}}}\mathrm{.}\end{array}$$

The upper bound of ${e}_{11}^{\lambda }$ can be obtained with

12 $${e}_{11}^{\lambda }\leqslant \overline{{e}_{11}^{\lambda }}=({Q}_{{c}_{1}{c}_{1}}^{\lambda }{E}_{{c}_{1}{c}_{1}}^{\lambda }-{q}_{\mathrm{0,}a}^{{c}_{1}}{Q}_{0{c}_{1}}^{\lambda }{E}_{0{c}_{1}}^{\lambda }-{q}_{\mathrm{0,}b}^{{c}_{1}}{Q}_{{c}_{1}0}^{\lambda }{E}_{{c}_{1}0}^{\lambda }-{q}_{\mathrm{0,}a}^{{c}_{1}}{q}_{\mathrm{0,}b}^{{c}_{1}}{Q}_{00}^{\lambda }{E}_{00}^{\lambda })/({q}_{\mathrm{1,}a}^{{c}_{1}}{q}_{\mathrm{1,}b}^{{c}_{1}}\underline{{Y}_{11}^{\lambda }}).$$

The subscripts *c*_0_ and *c*_1_ denote Alice or Bob prepare a signal state and a decoy state, respectively. If a subscript 0 appears, then Alice or Bob prepares a vacuum state.

To analyse the security and performance of our passive decoy state MDI-QKD, we still need to know the total gains and the overall error rates in both the *X* basis and the *Z* basis. Supplementary Material shows the calculating process that how to get the total gain and overall error rate theoretically.

### Passive Decoy State MDI-QKD With Intensity Fluctuation

We discuss an unavoidable imperfect factor, intensity fluctuation, in practice QKD protocol. We introduce parameter *δ* to denote the degree of intensity fluctuation. Here we take Alice as an example to describe the general process. The fluctuation ranges of the two intensities of Alice’s WCP sources are characterized by

13 $${\mu }_{1}\mathrm{(1}-{\delta }_{{\mu }_{1}})\leqslant {\mu }_{1}^{real}\leqslant {\mu }_{1}\mathrm{(1}+{\delta }_{{\mu }_{1}}),\quad {\mu }_{1}\mathrm{(1}-{\delta }_{{\mu }_{2}})\leqslant {\mu }_{1}^{real}\leqslant {\mu }_{1}\mathrm{(1}+{\delta }_{{\mu }_{2}}),$$

where ${\delta }_{{\mu }_{1}}$ and ${\delta }_{{\mu }_{2}}$ are the variation ranges of *μ*_1_ and *μ*_2_, respectively. ${\mu }_{1}^{real}$ and ${\mu }_{2}^{real}$ are the real intensities of Alice’s WCP sources. We assume that the range of the intensity fluctuation parameters ${\delta }_{{\mu }_{1}}$ and ${\delta }_{{\mu }_{2}}$ is $[0,\,0.1]$ ^34^.

Similarly, we can get

14 $${q}_{n,a}^{t,L}\leqslant {q}_{n,a}^{t,real}\leqslant {q}_{n,a}^{t,U},\quad {q}_{n,a}^{{c}_{0},L}\leqslant {q}_{n,a}^{{c}_{0},real}\leqslant {q}_{n,a}^{{c}_{0},U},\quad {q}_{n,a}^{{c}_{1},L}\leqslant {q}_{n,a}^{{c}_{1},real}\leqslant {q}_{n,a}^{{c}_{1},U},$$

where ${q}_{n,a}^{t,real}$ is the real total probability of having *n* photons in Alice’s output light, ${q}_{n,a}^{{c}_{0},real}$ and ${q}_{n,a}^{{c}_{1},real}$ are the joint probability of having *n* photons in mode *a* and no click or a click in the detector *a*_0_, respectively. The capital letter *L* and *U* represent the lower and the upper bounds.

Due to the intensity fluctuation, we can derive the following expressions:

15 $$\begin{array}{c}{q}_{\mathrm{0,}a}^{t,L}={I}_{\mathrm{0,}{\xi }_{a}^{U}}{e}^{-{\omega }_{a}^{U}},{q}_{\mathrm{1,}a}^{t,L}=({\omega }_{a}^{L}{I}_{\mathrm{0,}{\xi }_{a}^{L}}-{\xi }_{a}^{L}{I}_{\mathrm{1,}{\xi }_{a}^{L}}){e}^{-{\omega }_{a}^{L}},\\ {q}_{\mathrm{1,}a}^{t,U}=({\omega }_{a}^{U}{I}_{\mathrm{0,}{\xi }_{a}^{U}}-{\xi }_{a}^{U}{I}_{\mathrm{1,}{\xi }_{a}^{U}}){e}^{-{\omega }_{a}^{U}},{q}_{\mathrm{0,}a}^{t,U}={I}_{\mathrm{0,}{\xi }_{a}^{L}}{e}^{-{\omega }_{a}^{L}},\\ {q}_{\mathrm{0,}a}^{{c}_{0},L}={\tau }_{a}^{U}{I}_{\mathrm{0,(1}-{\eta }_{d}){\xi }_{a}^{U}},{q}_{\mathrm{0,}a}^{{c}_{0},U}={\tau }_{a}^{L}{I}_{\mathrm{0,(1}-{\eta }_{d}){\xi }_{a}^{L}},\\ {q}_{\mathrm{1,}a}^{{c}_{0},L}={\tau }_{a}^{L}({\omega }_{a}^{L}{I}_{\mathrm{0,(1}-{\eta }_{d}){\xi }_{a}^{L}}-{\xi }_{a}^{L}{I}_{\mathrm{1,(1}-{\eta }_{d}){\xi }_{a}^{L}}),\\ {q}_{\mathrm{1,}a}^{{c}_{0},U}={\tau }_{a}^{U}({\omega }_{a}^{U}{I}_{\mathrm{0,(1}-{\eta }_{d}){\xi }_{a}^{U}}-{\xi }_{a}^{U}{I}_{\mathrm{1,(1}-{\eta }_{d}){\xi }_{a}^{U}})\mathrm{.}\end{array}$$

Then, we have

16 $$\begin{array}{l}{q}_{\mathrm{0,}a}^{{c}_{1},L}={q}_{\mathrm{0,}a}^{t,L}-{q}_{\mathrm{0,}a}^{{c}_{0},L},\,{q}_{\mathrm{0,}a}^{{c}_{1},U}={q}_{\mathrm{0,}a}^{t,U}-{q}_{\mathrm{0,}a}^{{c}_{0},U},\,{q}_{\mathrm{1,}a}^{{c}_{1},L}={q}_{\mathrm{1,}a}^{t,L}-{q}_{\mathrm{1,}a}^{{c}_{0},L},\,{q}_{\mathrm{1,}a}^{{c}_{1},U}={q}_{\mathrm{1,}a}^{t,U}-{q}_{\mathrm{1,}a}^{{c}_{0},U},\end{array}$$

where

17 $$\begin{array}{ll}{\omega }_{a}^{L}\,=\,\frac{1}{2}[{\mu }_{1}\mathrm{(1}-{\delta }_{{\mu }_{1}})+{\mu }_{2}\mathrm{(1}-{\delta }_{{\mu }_{2}})], & {\omega }_{a}^{U}\,=\,\frac{1}{2}[{\mu }_{1}\mathrm{(1}+{\delta }_{{\mu }_{1}})+{\mu }_{2}\mathrm{(1}+{\delta }_{{\mu }_{2}})],\\ {\xi }_{a}^{L}\,=\,\sqrt{{\mu }_{1}\mathrm{(1}-{\delta }_{{\mu }_{1}}){\mu }_{2}\mathrm{(1}-{\delta }_{{\mu }_{2}})}, & {\xi }_{a}^{U}\,=\,\sqrt{{\mu }_{1}\mathrm{(1}+{\delta }_{{\mu }_{1}}){\mu }_{2}\mathrm{(1}+{\delta }_{{\mu }_{2}})},\\ {\tau }_{a}^{L}\,=\,\mathrm{(1}-\epsilon ){e}^{-[{\eta }_{d}{\mu }^{L}+\mathrm{(1}-{\eta }_{d}){\omega }_{a}^{L}]}, & {\tau }_{a}^{U}\,=\,\mathrm{(1}-\epsilon ){e}^{-[{\eta }_{d}{\mu }^{U}+\mathrm{(1}-{\eta }_{d}){\omega }_{a}^{U}]},\\ {\mu }^{L}\,=\,{\mu }_{1}\mathrm{(1}-{\delta }_{{\mu }_{1}})+{\mu }_{2}\mathrm{(1}-{\delta }_{{\mu }_{2}}), & {\mu }^{U}\,=\,{\mu }_{1}\mathrm{(1}+{\delta }_{{\mu }_{1}})+{\mu }_{2}\mathrm{(1}+{\delta }_{{\mu }_{2}}\mathrm{).}\end{array}$$

Bob has the same process as Alice. Next, we will calculate the lower bound of ${Q}_{11}^{{c}_{0}{c}_{0}}$ and ${Q}_{11}^{{c}_{1}{c}_{1}}$, i.e., ${Q}_{11}^{{c}_{0}{c}_{0},L}$ and ${Q}_{11}^{{c}_{1}{c}_{1},L}$, the upper bound of *e*_11_, i.e., ${e}_{11}^{U}$, when we consider the intensity fluctuation.

The overall gain of Alice’s and Bob’s detector both producing a click is ${Q}_{{c}_{1}{c}_{1}}$ and no click is ${Q}_{{c}_{0}{c}_{0}}$. They can be expressed as

18 $$\begin{array}{rcl}{Q}_{{c}_{0}{c}_{0}}=\sum _{n=0}^{\infty }\,\sum _{m=0}^{\infty }\,{Q}_{nm}^{{c}_{0}{c}_{0}} & = & {Q}_{00}^{{c}_{0}{c}_{0}}+{Q}_{11}^{{c}_{0}{c}_{0}}+\sum _{m=1}^{\infty }\,{Q}_{0m}^{{c}_{0}{c}_{0}}+\sum _{n=1}^{\infty }\,{Q}_{n0}^{{c}_{0}{c}_{0}}\\  &  & +\sum _{m=2}^{\infty }\,{Q}_{1m}^{{c}_{0}{c}_{0}}+\sum _{n=2}^{\infty }\,{Q}_{n1}^{{c}_{0}{c}_{0}}+\sum _{n=2}^{\infty }\,\sum _{m=2}^{\infty }\,{Q}_{nm}^{{c}_{0}{c}_{0}},\\ {Q}_{{c}_{1}{c}_{1}}=\sum _{n=0}^{\infty }\,\sum _{m=0}^{\infty }\,{Q}_{nm}^{{c}_{1}{c}_{1}} & = & {Q}_{00}^{{c}_{1}{c}_{1}}+{Q}_{11}^{{c}_{1}{c}_{1}}+\sum _{m=1}^{\infty }\,{Q}_{0m}^{{c}_{1}{c}_{1}}+\sum _{n=1}^{\infty }\,{Q}_{n0}^{{c}_{1}{c}_{1}}\\  &  & +\sum _{m=2}^{\infty }\,{Q}_{1m}^{{c}_{1}{c}_{1}}+\sum _{n=2}^{\infty }\,{Q}_{n1}^{{c}_{1}{c}_{1}}+\sum _{n=2}^{\infty }\,\sum _{m=2}^{\infty }\,{Q}_{nm}^{{c}_{1}{c}_{1}},\end{array}$$

where the parameters are in the condition of using ${\mu }_{1}^{real}$, ${\mu }_{2}^{real}$, ${\upsilon }_{1}^{real}$ and ${\upsilon }_{1}^{real}$.

Then, applying ${Q}_{{c}_{1}{c}_{1}}-{Q}_{{c}_{0}{c}_{0}}$, we can get

19 $$\begin{array}{rcl}{Q}_{11}^{{c}_{0}{c}_{0}}-{Q}_{11}^{{c}_{1}{c}_{1}} & = & {Q}_{{c}_{0}{c}_{0}}-{Q}_{{c}_{1}{c}_{1}}+{Q}_{00}^{{c}_{1}{c}_{1}}-{Q}_{00}^{{c}_{0}{c}_{0}}+\sum _{m=1}^{\infty }\,{Q}_{0m}^{{c}_{1}{c}_{1}}\\  &  & -\sum _{m=1}^{\infty }\,{Q}_{0m}^{{c}_{0}{c}_{0}}+\sum _{n=1}^{\infty }\,{Q}_{n0}^{{c}_{1}{c}_{1}}-\sum _{n=1}^{\infty }\,{Q}_{n0}^{{c}_{0}{c}_{0}}\\  &  & +\sum _{m=2}^{\infty }\,{Q}_{1m}^{{c}_{1}{c}_{1}}-\sum _{m=2}^{\infty }\,{Q}_{1m}^{{c}_{0}{c}_{0}}+\sum _{n=2}^{\infty }\,{Q}_{n1}^{{c}_{1}{c}_{1}}\\  &  & -\sum _{n=2}^{\infty }\,{Q}_{n1}^{{c}_{0}{c}_{0}}+\sum _{n=2}^{\infty }\,\sum _{m=2}^{\infty }\,{Q}_{nm}^{{c}_{1}{c}_{1}}-\sum _{n=2}^{\infty }\,\sum _{m=2}^{\infty }\,{Q}_{nm}^{{c}_{0}{c}_{0}}\mathrm{.}\end{array}$$

The common point between the passive decoy state and the active decoy state is that the counting rates and the error rates of pulse of the same photon number states from the signal states and the decoy states shall be equal to each other^37^. Thus, in our study, we assume they are still equal to each other in the case of intensity fluctuation. Then, we use the following inequalities to substitute the elements in Eq. (19):

20 $$\begin{array}{l}{Q}_{00}^{{c}_{0}{c}_{0}}\leqslant \frac{{q}_{\mathrm{0,}a}^{{c}_{0},U}{q}_{\mathrm{0,}b}^{{c}_{0},U}{Q}_{00}^{{c}_{1}{c}_{1}}}{{q}_{\mathrm{0,}a}^{{c}_{1},L}{q}_{\mathrm{0,}b}^{{c}_{0},L}},\quad {Q}_{nm}^{{c}_{0}{c}_{0}}\leqslant \frac{{q}_{n,a}^{{c}_{0},U}{q}_{m,b}^{{c}_{0},U}{Q}_{nm}^{{c}_{1}{c}_{1}}}{{q}_{n,a}^{{c}_{1},L}{q}_{m,b}^{{c}_{1},L}},\quad 0\le {Q}_{00}^{{c}_{1}{c}_{1}}\leqslant \frac{{E}_{{c}_{1}{c}_{1}}{Q}_{{c}_{1}{c}_{1}}}{{e}_{00}}\mathrm{.}\end{array}$$

And note that $n\geqslant 1$, we have ${q}_{n,a}^{{c}_{1},L}/{q}_{n,a}^{{c}_{0},U}\leqslant {q}_{\mathrm{1,}a}^{{c}_{1},L}/{q}_{\mathrm{1,}a}^{{c}_{0},U}$ and ${q}_{m,b}^{{c}_{1},U}/{q}_{m,b}^{{c}_{0},L}\leqslant {q}_{\mathrm{1,}b}^{{c}_{1},U}/{q}_{\mathrm{1,}b}^{{c}_{0},L}$. By using this inequalities, the elements in Eq. (19) can be substituted as:

21 $$\begin{array}{l}{Q}_{11}^{{c}_{0}{c}_{0}}-{Q}_{11}^{{c}_{1}{c}_{1}}\leqslant {Q}_{11}^{{c}_{1}{c}_{1}}(\tfrac{{q}_{\mathrm{1,}a}^{{c}_{0},U}{q}_{\mathrm{1,}b}^{{c}_{0},U}}{{q}_{\mathrm{1,}a}^{{c}_{1},L}{q}_{\mathrm{1,}b}^{{c}_{1},L}}-1),\\ {Q}_{00}^{{c}_{1}{c}_{1}}-{Q}_{00}^{{c}_{0}{c}_{0}}\geqslant -\tfrac{{E}_{{c}_{1}{c}_{1}}{Q}_{{c}_{1}{c}_{1}}}{{e}_{00}}(\tfrac{{q}_{\mathrm{0,}a}^{{c}_{0},U}{q}_{\mathrm{0,}b}^{{c}_{0},U}}{{q}_{\mathrm{0,}a}^{{c}_{1},L}{q}_{\mathrm{0,}b}^{{c}_{1},L}}-1),\\ \sum _{m=1}^{\infty }\,{Q}_{0m}^{{c}_{1}{c}_{1}}-\sum _{m=1}^{\infty }\,{Q}_{0m}^{{c}_{0}{c}_{0}}\geqslant \sum _{m=1}^{\infty }\,-{Q}_{0m}^{{c}_{1}{c}_{1}}(\tfrac{{q}_{\mathrm{0,}a}^{{c}_{0},U}{q}_{\mathrm{1,}b}^{{c}_{0},U}}{{q}_{\mathrm{0,}a}^{{c}_{1},L}{q}_{\mathrm{1,}b}^{{c}_{1},L}}-1),\\ \sum _{n=1}^{\infty }\,{Q}_{n0}^{{c}_{1}{c}_{1}}-\sum _{n=1}^{\infty }\,{Q}_{n0}^{{c}_{0}{c}_{0}}\geqslant \sum _{n=1}^{\infty }\,-{Q}_{n0}^{{c}_{1}{c}_{1}}(\tfrac{{q}_{\mathrm{1,}a}^{{c}_{0},U}{q}_{\mathrm{0,}b}^{{c}_{0},U}}{{q}_{\mathrm{1,}a}^{{c}_{1},L}{q}_{\mathrm{0,}b}^{{c}_{1},L}}-1),\\ \sum _{m=2}^{\infty }\,{Q}_{1m}^{{c}_{1}{c}_{0}}-\sum _{m=2}^{\infty }\,{Q}_{1m}^{{c}_{0}{c}_{0}}\geqslant \sum _{m=2}^{\infty }\,-{Q}_{1m}^{{c}_{1}{c}_{1}}(\tfrac{{q}_{\mathrm{1,}a}^{{c}_{0},U}{q}_{\mathrm{1,}b}^{{c}_{0},U}}{{q}_{\mathrm{1,}a}^{{c}_{1},L}{q}_{\mathrm{1,}b}^{{c}_{1},L}}-1),\\ \sum _{n=2}^{\infty }\,{Q}_{n1}^{{c}_{1}{c}_{1}}-\sum _{n=2}^{\infty }\,{Q}_{n1}^{{c}_{0}{c}_{0}}\geqslant \sum _{n=2}^{\infty }\,-{Q}_{n1}^{{c}_{1}{c}_{1}}(\tfrac{{q}_{\mathrm{1,}a}^{{c}_{0},U}{q}_{\mathrm{1,}b}^{{c}_{0},U}}{{q}_{\mathrm{1,}a}^{{c}_{1},L}{q}_{\mathrm{1,}b}^{{c}_{1},L}}-1),\\ \sum _{n=2}^{\infty }\,\sum _{m=2}^{\infty }\,{Q}_{nm}^{{c}_{1}{c}_{1}}-\sum _{n=2}^{\infty }\,\sum _{m=2}^{\infty }\,{Q}_{nm}^{{c}_{0}{c}_{0}}\geqslant \sum _{n=2}^{\infty }\,\sum _{m=2}^{\infty }\,-{Q}_{nm}^{{c}_{1}{c}_{1}}(\tfrac{{q}_{\mathrm{1,}a}^{{c}_{0},U}{q}_{\mathrm{1,}b}^{{c}_{0},U}}{{q}_{\mathrm{1,}a}^{{c}_{1},L}{q}_{\mathrm{1,}b}^{{c}_{1},L}}-1)\mathrm{.}\end{array}$$

Finally, we obtain the lower bound of ${Q}_{11}^{{c}_{1}{c}_{1}}$,

22 $${Q}_{11}^{{c}_{1}{c}_{1}}\ge {Q}_{11}^{{c}_{1}{c}_{1},L}=\frac{{Q}_{{c}_{0}{c}_{0}}-{Q}_{{c}_{1}{c}_{1}}-(\frac{{q}_{\mathrm{0,}a}^{{c}_{0},U}{q}_{\mathrm{0,}b}^{{c}_{0},U}}{{q}_{\mathrm{0,}a}^{{c}_{1},L}{q}_{\mathrm{0,}b}^{{c}_{1},L}}-1)\frac{{E}_{{c}_{1}{c}_{1}}{Q}_{{c}_{1}{c}_{1}}}{{e}_{00}}}{\frac{{q}_{\mathrm{1,}a}^{{c}_{0},U}{q}_{\mathrm{1,}b}^{{c}_{0},U}}{{q}_{\mathrm{1,}a}^{{c}_{1},L}{q}_{\mathrm{1,}b}^{{c}_{1},L}}-1}.$$

We can get

23 $$\frac{{Q}_{11}^{{c}_{1}{c}_{1}}}{{q}_{\mathrm{1,}a}^{{c}_{1}}{q}_{\mathrm{1,}b}^{{c}_{1}}}={Y}_{11}^{{c}_{0}{c}_{0}}={Y}_{11}^{{c}_{0}{c}_{0}}=\frac{{Q}_{11}^{{c}_{0}{c}_{0}}}{{q}_{\mathrm{1,}a}^{{c}_{0}}{q}_{\mathrm{1,}b}^{{c}_{0}}},\quad {Q}_{11}^{{c}_{0}{c}_{0},L}=\frac{{Q}_{11}^{{c}_{1}{c}_{1},L}{q}_{\mathrm{1,}a}^{{c}_{0},L}{q}_{\mathrm{1,}b}^{{c}_{0},L}}{{q}_{\mathrm{1,}a}^{{c}_{0},U}{q}_{\mathrm{1,}b}^{{c}_{1},U}}.$$

Then, we will calculate the upper bound of *e*_11_. The overall quantum bit error rate (QBER) is

24 $$\begin{array}{rcl}{E}_{{c}_{1}{c}_{1}}{Q}_{{c}_{1}{c}_{1}} & = & {q}_{\mathrm{0,}a}^{{c}_{1}}{q}_{\mathrm{0,}b}^{{c}_{1}}{e}_{00}{Y}_{00}+{q}_{\mathrm{1,}a}^{{c}_{1}}{q}_{\mathrm{1,}b}^{{c}_{1}}{e}_{11}{Y}_{11}+{q}_{\mathrm{0,}a}^{{c}_{1}}\sum _{m=1}^{\infty }\,{q}_{m,b}^{{c}_{1}}{e}_{0m}{Y}_{0m}+{q}_{\mathrm{0,}b}^{{c}_{1}}\sum _{n=1}^{\infty }\,{q}_{n,a}^{{c}_{1}}{e}_{n0}{Y}_{n0}\\  &  & +{q}_{\mathrm{1,}a}^{{c}_{1}}\sum _{m=2}^{\infty }\,{q}_{m,b}^{{c}_{1}}{e}_{1m}{Y}_{1m}+{q}_{\mathrm{1,}b}^{{c}_{1}}\sum _{n=2}^{\infty }\,{q}_{n,a}^{{c}_{1}}{e}_{n1}{Y}_{n1}+\sum _{n,m=2}^{\infty }\,{q}_{n,a}^{{c}_{1}}{q}_{n,b}^{{c}_{1}}{e}_{nm}{Y}_{nm}\\  & \ge  & {q}_{\mathrm{0,}a}^{{c}_{1}}{Q}_{0{c}_{1}}{E}_{0{c}_{1}}+{q}_{\mathrm{0,}a}^{{c}_{1}}{q}_{\mathrm{0,}b}^{{c}_{1}}{e}_{11}{Y}_{11}+{q}_{\mathrm{0,}b}^{{c}_{1}}{Q}_{{c}_{1}0}{E}_{{c}_{1}0}+{q}_{\mathrm{0,}a}^{{c}_{1}}{q}_{\mathrm{0,}b}^{{c}_{1}}{Q}_{00}{E}_{00}\mathrm{.}\end{array}$$

we can obtain the upper bound of *e*_11_

25 $${e}_{11}\le {e}_{11}^{U}=\frac{1}{{Q}_{11}^{{c}_{1}{c}_{1},L}}({Q}_{{c}_{1}{c}_{1}}{E}_{{c}_{1}{c}_{1}}-{q}_{\mathrm{0,}a}^{{c}_{1}}{Q}_{0{c}_{1}}{E}_{0{c}_{1}}-{q}_{\mathrm{0,}b}^{{c}_{1}}{Q}_{{c}_{1}0}{E}_{{c}_{1}0}-{q}_{\mathrm{0,}a}^{{c}_{1}}{q}_{\mathrm{0,}b}^{{c}_{1}}{Q}_{00}{E}_{00}\mathrm{).}$$

The key generation rate with intensity fluctuation is

26 $$R\ge ({Q}_{11}^{{c}_{1}{c}_{1},L}+{Q}_{11}^{{c}_{0}{c}_{0},L})\,[1-H({e}_{11}^{U})]-{Q}_{{c}_{0}{c}_{0}}{f}_{e}({E}_{{c}_{0}{c}_{0}})\,H({E}_{{c}_{0}{c}_{0}})\mathrm{.}$$

### Statistical Fluctuation

In practical, the number of key distribution is finite, which will bring some statistical fluctuation into the parameter estimation. In this section, we will discuss the effect of the finite size on the security of MDI-QKD with our passive decoy state method based on the standard statistical analysis^38, 39^.

When consider the statistical fluctuation, the total gain ${Q}_{{c}_{i}{c}_{j}}^{\lambda }$ and the overall error rate ${E}_{{c}_{i}{c}_{j}}^{\lambda }$ are turned from determined values into intervals, which can be written as

27 $$\underline{{Q}_{{c}_{i}{c}_{j}}^{\lambda }}\le {Q}_{{c}_{i}{c}_{j}}^{\lambda }\le \overline{{Q}_{{c}_{i}{c}_{j}}^{\lambda }},\quad \underline{{Q}_{{c}_{i}{c}_{j}}^{\lambda }{E}_{{c}_{i}{c}_{j}}^{\lambda }}\le {Q}_{{c}_{i}{c}_{j}}^{\lambda }{E}_{{c}_{i}{c}_{j}}^{\lambda }\le \overline{{Q}_{{c}_{i}{c}_{j}}^{\lambda }{E}_{{c}_{i}{c}_{j}}^{\lambda }},$$

where

28 $$\begin{array}{c}\quad \quad \underline{{Q}_{{c}_{i}{c}_{j}}^{\lambda }}={Q}_{{c}_{i}{c}_{j}}^{\lambda }\mathrm{(1}-{\gamma }_{1}),\overline{{Q}_{{c}_{i}{c}_{j}}^{\lambda }}={Q}_{{c}_{i}{c}_{j}}^{\lambda }\mathrm{(1}+{\gamma }_{1}),\\ \quad \quad \quad {\gamma }_{1}={\sigma }_{\alpha }/\sqrt{{N}_{{c}_{i}{c}_{j}}^{\lambda }{Q}_{{c}_{i}{c}_{j}}^{\lambda }},{\gamma }_{2}={\sigma }_{\alpha }/\sqrt{{N}_{{c}_{i}{c}_{j}}^{\lambda }{Q}_{{c}_{i}{c}_{j}}^{\lambda }{E}_{{c}_{i}{c}_{j}}^{\lambda }},\\ \,\,\,\underline{{Q}_{{c}_{i}{c}_{j}}^{\lambda }{E}_{{c}_{i}{c}_{j}}^{\lambda }}={Q}_{{c}_{i}{c}_{j}}^{\lambda }{E}_{{c}_{i}{c}_{j}}^{\lambda }\mathrm{(1}-{\gamma }_{2}),\overline{{Q}_{{c}_{i}{c}_{j}}^{\lambda }{E}_{{c}_{i}{c}_{j}}^{\lambda }}={Q}_{{c}_{i}{c}_{j}}^{\lambda }{E}_{{c}_{i}{c}_{j}}^{\lambda }\mathrm{(1}+{\gamma }_{2}\mathrm{).}\end{array}$$

Here *σ*_*α*_ is the number of standard deviations, which is related to the failure probability of the security analysis. we choose *σ*_*α*_ = 5, which means the failure probability is 5.73 × 10^7^. These parameters used in our method are the same as those in the refs 12 and 29. ${N}_{{c}_{i}{c}_{j}}^{\lambda }$ is the length of data in the situation that Alice has the *c*_*i*_ mode and Bob has the *c*_*j*_ mode, where *i*,*j* = 0 or 1. Thus, the lower bound of ${Y}_{11}^{\lambda }$ and the upper bound of ${e}_{11}^{\lambda }$ given by Eqs (9) and (12), respectively, can be modified to ref. 29

29 $$\begin{array}{rcl}{Y}_{11}^{\lambda }\ge \underline{\underline{{Y}_{11}^{\lambda }}} & = & ({c}_{{c}_{1}{c}_{1}}\overline{{Q}_{{c}_{1}{c}_{1}}^{\lambda }}+{c}_{{c}_{1}{c}_{0}}\underline{{Q}_{{c}_{1}{c}_{0}}^{\lambda }}+{c}_{{c}_{0}{c}_{1}}\underline{{Q}_{{c}_{0}{c}_{1}}^{\lambda }}+{c}_{{c}_{0}{c}_{0}}\overline{{Q}_{{c}_{0}{c}_{0}}^{\lambda }}\\  &  & -{c}_{{c}_{1}0}\overline{{Q}_{{c}_{1}0}^{\lambda }}-{c}_{{c}_{0}0}\overline{{Q}_{{c}_{0}0}^{\lambda }}-{c}_{0{c}_{1}}\overline{{Q}_{0{c}_{1}}^{\lambda }}-{c}_{0{c}_{0}}\overline{{Q}_{0{c}_{0}}^{\lambda }}+{c}_{00}\underline{{Q}_{00}^{\lambda }})\\  &  & \times {[(K-{A}_{1}{B}_{1})({q}_{\mathrm{0,}a}^{{c}_{0}}{q}_{\mathrm{1,}a}^{{c}_{0}}+{q}_{\mathrm{0,}a}^{{c}_{1}}{q}_{\mathrm{1,}a}^{{c}_{1}})({q}_{\mathrm{0,}b}^{{c}_{0}}{q}_{\mathrm{1,}b}^{{c}_{0}}+{q}_{\mathrm{0,}b}^{{c}_{1}}{q}_{\mathrm{1,}b}^{{c}_{1}})]}^{-1},\end{array}$$

30 $${e}_{11}^{\lambda }\le \overline{\overline{{e}_{11}^{\lambda }}}=(\overline{{Q}_{{c}_{1}{c}_{1}}^{\lambda }{E}_{{c}_{1}{c}_{1}}^{\lambda }}-{q}_{\mathrm{0,}a}^{{c}_{1}}\underline{{Q}_{0{c}_{1}}^{\lambda }{E}_{0{c}_{1}}^{\lambda }}-{q}_{\mathrm{0,}b}^{{c}_{1}}\underline{{Q}_{{c}_{1}0}^{\lambda }{E}_{{c}_{1}0}^{\lambda }}-{q}_{\mathrm{0,}a}^{{c}_{1}}{q}_{\mathrm{0,}b}^{{c}_{1}}\underline{{Q}_{00}^{\lambda }{E}_{00}^{\lambda }})/({q}_{\mathrm{1,}a}^{{c}_{1}}{q}_{\mathrm{1,}b}^{{c}_{1}}\underline{{Y}_{11}^{\lambda }}).$$

We can also modify the lower bound of ${Q}_{11}^{{c}_{1}{c}_{1}}$ and the upper bound of ${e}_{11}^{\lambda }$ given by Eqs (22) and (25), respectively, as follows

31 $${Q}_{11}^{{c}_{1}{c}_{1}}\ge \underline{{Q}_{11}^{{c}_{1}{c}_{1},L}}=\frac{\underline{{Q}_{{c}_{0}{c}_{0}}}-\overline{{Q}_{{c}_{1}{c}_{1}}}-(\frac{{q}_{\mathrm{0,}a}^{{c}_{0},U}{q}_{\mathrm{0,}b}^{{c}_{0},U}}{{q}_{\mathrm{0,}a}^{{c}_{1},L}{q}_{\mathrm{0,}b}^{{c}_{1},L}}-1)\frac{\underline{{E}_{{c}_{1}{c}_{1}}{Q}_{{c}_{1}{c}_{1}}}}{{e}_{00}}}{\frac{{q}_{\mathrm{1,}a}^{{c}_{0},U}{p}_{\mathrm{1,}b}^{{c}_{0},U}}{{q}_{\mathrm{1,}a}^{{c}_{1},L}{q}_{\mathrm{1,}b}^{{c}_{1},L}}-1},$$

32 $${e}_{11}\le \overline{{e}_{11}^{U}}=\frac{1}{\underline{{Q}_{11}^{{c}_{1}{c}_{1},L}}}(\overline{{Q}_{{c}_{1}{c}_{1}}{E}_{{c}_{1}{c}_{1}}}-{q}_{\mathrm{0,}a}^{{c}_{1}}\underline{{Q}_{0{c}_{1}}{E}_{0{c}_{1}}}-{q}_{\mathrm{0,}b}^{{c}_{1}}\underline{{Q}_{{c}_{1}0}{E}_{{c}_{1}0}}-{q}_{\mathrm{0,}a}^{{c}_{1}}{q}_{\mathrm{0,}b}^{{c}_{1}}\underline{{Q}_{00}{E}_{00}}).$$

Substituting Eqs (29) and (30) into Eqs (7), (31) and (32) into Eq. (26), we can respectively estimate the key generation rate with or without intensity fluctuation in the case of finite resource in different data length. In our method, we assume that Alice’s and Bob’s data length are the same for each pair of intensities.

### Numerical Simulation

**Figure 2:**
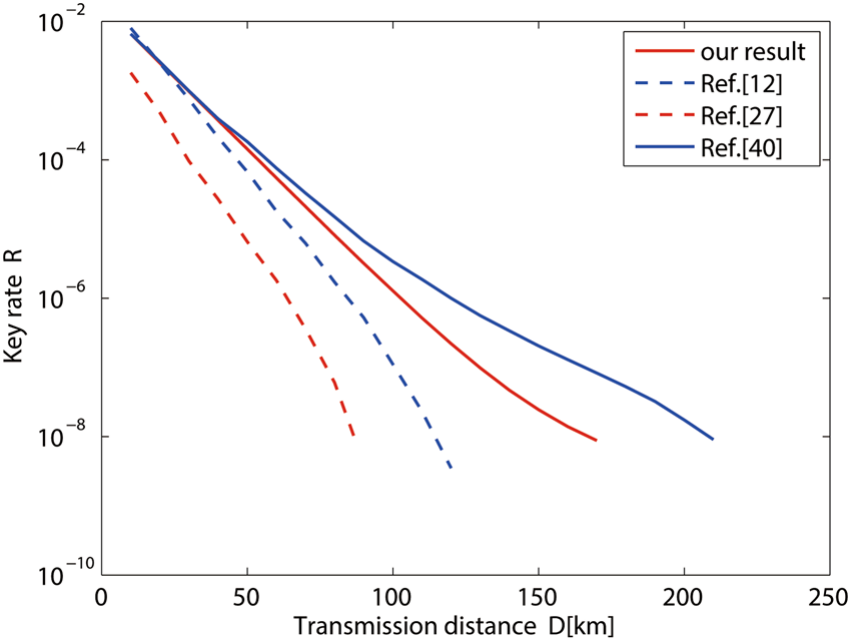
Key generation rate versus the total transmission distance with the passive decoy state method based on polarization encoding mode (red solid line) compared to the passive decoy state method based on phase encoding mode (red dot-dashed line; ref. 29), the active decoy state method using two decoy states (blue dot-dashed line; ref. 12), and recently optimal active decoy state method (blue solid line; ref. 17).

From our security analysis, we can obtain the yield ${Y}_{11}^{Z}$ and the error rate ${e}_{11}^{X}$, respectively, when Alice and Bob send single-photon pulses to Charles, as well as the total gains and the overall error rates in both the *X* basis and the *Z* basis. Then, we can get the key generation rate plotted in Fig. 2. The practical parameters for numerical simulations used in our method are *η*_*d*_ = 14.5%, *e*_*d*_ = 1.5%, *Y*_0_ = 3 × 10^−6^, *f*_*e*_ = 1.16 and *α* = 0.2 *dB*/*km*. These experimental parameters, including the detection efficiency *η*_*d*_, the total misalignment error *e*_*d*_ and the background rate *Y*_0_, are from the 144 km QKD experiment reported in ref. 40. Since two PDs (Photon Detectors) are used in ref. 40, the background rate of each PD here is roughly a quarter of the value there. We assume our model that the six PDs in MDI-QKD (see Fig. 1) have identical *η*_*d*_ and *Y*_0_.

In Fig. 2, we compare the key generation rate of MDI-QKD given by our passive decoy state method with that given by an active decoy state method with two decoy states in ref. 12 and recently optimal active decoy state method in ref. 17. The key generation rate is maximized by optimizing the intensity of sources. It can clearly be seen that the passive decoy state method can provide a performance comparable to the active one. We also compare the key generation rate of MDI-QKD given by our passive decoy state method which based on polarization encoding mode with that based on phase encoding mode in ref. 29, due to these two encoding modes are both applied in practical systems.

**Figure 3:**
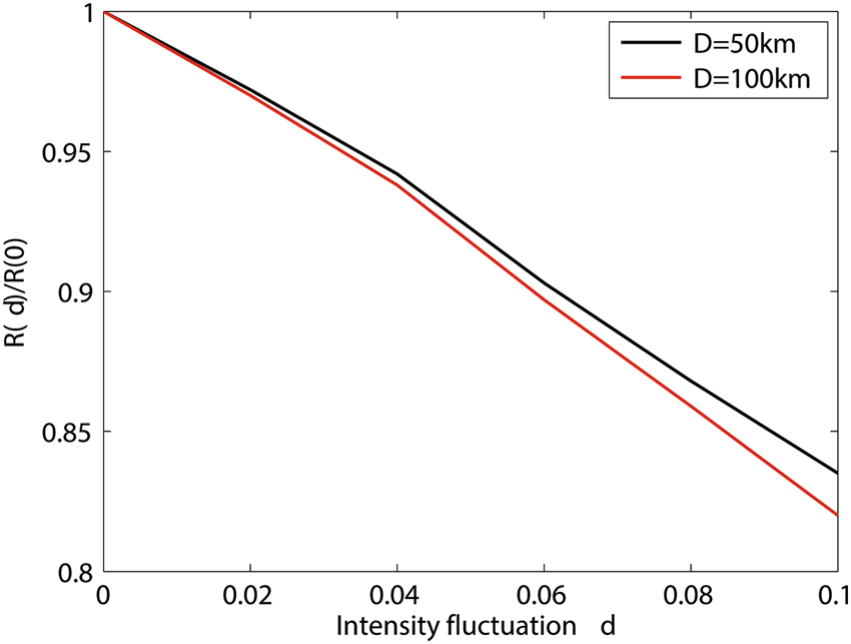
The fidelity of the the key generation rate *R*(*δ*)/*R*(0) versus intensity fluctuation *δ*.

In addition, we will characterize the relationship between the key generation rate and the intensity fluctuation when transmission distance *d* is fixed. The result is shown in Fig. 3. Define *R*(*δ*)/*R*(0) as the fidelity of the the key generation rate with passive decoy state method, where *R*(*δ*) denotes the the key generation rate *R* with intensity fluctuation and *R*(0) denotes the the key generation rate *R* with no intensity fluctuation. From Fig. 3, we can see that the *R*(*δ*)/*R*(0) is getting to 0 with *δ* getting to 0.1. It indicates that when intensity fluctuation increases, the fidelity decreases, so does the key generation rate. Furthermore, we can also get that the effect of intensity fluctuation on the key generation rate monotonously increases with the increase of the transmission distance. So when we analyse the performance of MDI-QKD, the influence of intensity fluctuation can not be neglected, especially over long-distance communications.

**Figure 4:**
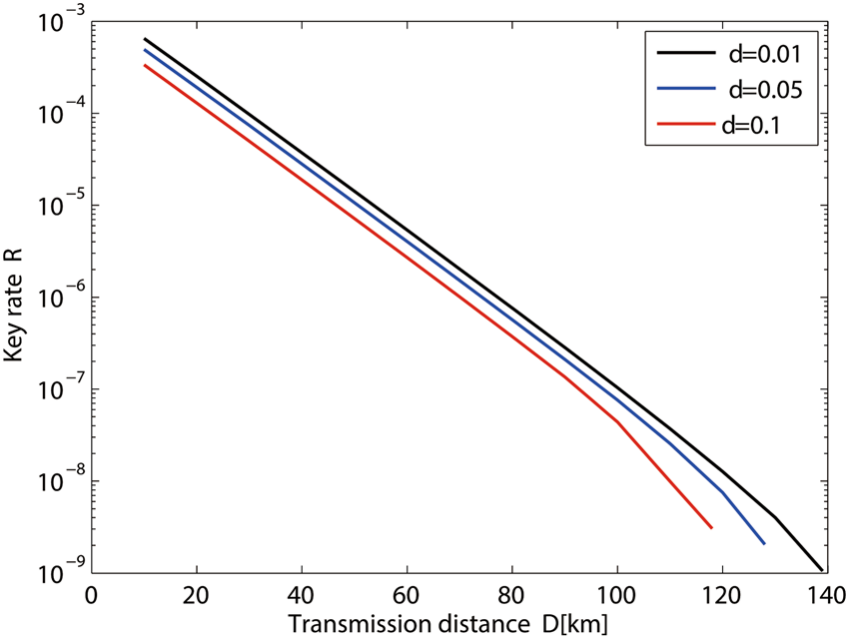
Secret key rate *R* versus the transmission distance with *δ* = 0.01,0.05,0.09,0.1 (curves from right to left).

**Figure 5:**
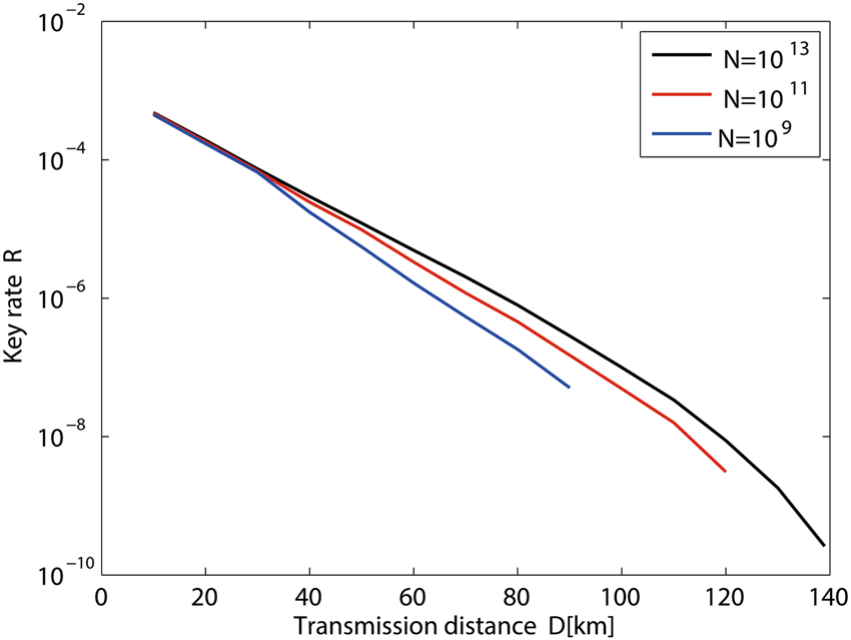
Secret key rate *R* versus the transmission distance for *δ* = 0.05 and *N* = 1 × 10^*x*^ with *x* = 9,10,11,12,13 (curves from left to right).

Figure 4 shows the key generation rate of MDI-QKD given by our passive-decoy-state method with different intensity fluctuation. We can find that intensity fluctuation obviously limit the secret key rate. In order to further study the effect of intensity fluctuation for different total numbers of transmitting signals *N*, we show the relations between *R*(*δ*)/*R*(0) and the secure transmission distance given that the intensity fluctuation is fixed to be 0.05 in Fig. 5. We can find that the smaller the data size of total transmitting signals is, more obvious the effect of intensity fluctuation is.

## Discussion

In conclusion, we applied the passive decoy state method in the MDI-QKD based on polarization encoding mode, and gave a security analysis of this protocol. Using the passive decoy state method, not only all detector side channel attacks can be removed, but also side channel attacks on the sources can be overcome, which the active source modulation method may bring. We analysed the security of this protocol, and found that the MDI-QKD with our passive decoy state method can have a performance comparable to the protocol with the active decoy state method and the passive decoy state method based on phase encoding mode. To fit for the demand of practical application, we discuss intensity fluctuation in the security analysis of passive decoy state MDI-QKD protocol. In this case, we got the key generation rate through the formulas of yield and error rate derived in our paper. Based on the total gain and the overall error rate derived in our paper, we gave numerical simulations for our protocol. We showed that intensity fluctuation has a non-negligible effect on the secret key rate of the passive decoy state MDI-QKD protocol, especially in the case of small data size of total transmitting signals and long distance transmission. In addition, our analysis of statistical fluctuation shows that the finite-size effect also limits the key generation rate of MDI-QKD with passive decoy state method.

## Electronic supplementary material

Supplementary Information
