# Supplementary material for: Practical passive decoy state measurement-device-independent quantum key distribution with unstable sources
Source: Sci Rep. 2017 Sep 12;7:11370. doi: 10.1038/s41598-017-09367-y (PMC5595959; doi:10.1038/s41598-017-09367-y)
Supplement: Supplementary file 1 — Supplementary Information [file 41598_2017_9367_MOESM1_ESM.pdf]

Practical passive decoy state measurement-device-independent quantum key distribution with unstable sources

Li Liu, Fen-Zhuo Guo, Qiao-Yan Wen

Supplementary Material

We give the calculating process that how to get the total gain and overall error rate theoretically.

First, Alice and Bob phase-randomized WCPs to prepare coherent states with intensities of μ_1, μ_2, υ_1, and υ_2 respectively. We can get the Alice and Bobs joint state,

$$\left|e^{i\phi_{a_1}}\sqrt{\mu_1}\right\rangle_{a_1}\left|e^{i\phi_{a_2}}\sqrt{\mu_2}\right\rangle_{a_2}\left|e^{i\phi_{b_1}}\sqrt{\upsilon_1}\right\rangle_{b_1}\left|e^{i\phi_{b_2}}\sqrt{\upsilon_2}\right\rangle_{b_2}, \tag{1}$$

where ϕ_{a_1}, ϕ_{a_2}, ϕ_{b_1}, and ϕ_{b_2} are randomized phases. Two sources on both Alice's and Bob's side interfere at a balanced BS, and one output reaches the detector while the other transmits through a lossy channel to Charles. Thus, the joint state can be expressed by

$$\begin{aligned}&\left|e^{i\phi_{a_1}}\sqrt{\frac{\mu_1}{2}}+e^{i\phi_{a_2}}\sqrt{\frac{\mu_2}{2}}\right\rangle_a\left|e^{i\phi_{a_1}}\sqrt{\frac{\mu_1}{2}}-e^{i\phi_{a_2}}\sqrt{\frac{\mu_2}{2}}\right\rangle_{a_0}\left|e^{i\phi_{b_1}}\sqrt{\frac{\upsilon_1}{2}}+e^{i\phi_{b_2}}\sqrt{\frac{\upsilon_2}{2}}\right\rangle_b\\&\otimes\left|e^{i\phi_{b_1}}\sqrt{\frac{\upsilon_1}{2}}-e^{i\phi_{b_2}}\sqrt{\frac{\upsilon_2}{2}}\right\rangle_{b_0},\end{aligned} \tag{2}$$

where the subscripts a and b express the paths towards Charles, while a_0 and b_0 express the paths to detectors. After the pulses separately pass through two lossy channels (between Alice and Charles with transmissivity η_a, Bob and Charles with transmissivity η_b, the joint state can then be rewritten as

$$\begin{aligned}&\left|e^{i\phi_{a_1}}\sqrt{\frac{\mu_1}{2}}-e^{i\phi_{a_2}}\sqrt{\frac{\mu_2}{2}}\right\rangle_{a_0}\left|e^{i\phi_{b_1}}\sqrt{\frac{\upsilon_1}{2}}-e^{i\phi_{b_2}}\sqrt{\frac{\upsilon_2}{2}}\right\rangle_{b_0}\left|e^{i\phi_{a_1}}\sqrt{\frac{\mu_1\eta_a}{2}}+e^{i\phi_{a_2}}\sqrt{\frac{\mu_2\eta_a}{2}}\right\rangle_a\\&\otimes\left|e^{i\phi_{b_1}}\sqrt{\frac{\upsilon_1\eta_b}{2}}+e^{i\phi_{b_2}}\sqrt{\frac{\upsilon_2\eta_b}{2}}\right\rangle_b.\end{aligned} \tag{3}$$

Then the pulses on output a and b interfere at a balanced BS in Charless laboratory, labeled by c and d, we can get the joint state as follow form,

$$\begin{aligned}&\left|e^{i\phi_{a_1}}\sqrt{\frac{\mu_1}{2}}-e^{i\phi_{a_2}}\sqrt{\frac{\mu_2}{2}}\right\rangle_{a_0}\left|e^{i\phi_{b_1}}\sqrt{\frac{\upsilon_1}{2}}-e^{i\phi_{b_2}}\sqrt{\frac{\upsilon_2}{2}}\right\rangle_{b_0}\left|e^{i\phi_{a_1}}\sqrt{\frac{\mu_1\eta_a}{4}}+e^{i\phi_{a_2}}\sqrt{\frac{\mu_2\eta_a}{4}}+e^{i\phi_{b_1}}\sqrt{\frac{\upsilon_1\eta_b}{4}}\right.\\&\left.+e^{i\phi_{b_2}}\sqrt{\frac{\upsilon_2\eta_b}{4}}\right\rangle_c\left|e^{i\phi_{a_1}}\sqrt{\frac{\mu_1\eta_a}{4}}+e^{i\phi_{a_2}}\sqrt{\frac{\mu_2\eta_a}{4}}-e^{i\phi_{b_1}}\sqrt{\frac{\upsilon_1\eta_b}{4}}-e^{i\phi_{b_2}}\sqrt{\frac{\upsilon_2\eta_b}{4}}\right\rangle_d.\end{aligned} \tag{4}$$

After the PBS in Charless laboratory, the optical intensities received by each SPD are given by

$$\begin{aligned}D_{ch}:|A|^2=&(1-e_d)[\frac{\mu_1\eta_a+\mu_2\eta_a}{4}+\frac{\upsilon_1\eta_b+\upsilon_2\eta_b}{4}+\frac{\eta_a\sqrt{\mu_1\mu_2}}{2}cos(\phi_{a_2}-\phi_{a_1})+\frac{\sqrt{\mu_1\upsilon_1\eta_a\eta_b}}{2}cos(\phi_{b_1}-\phi_{a_1})\\&+\frac{\eta_b\sqrt{\upsilon_1\upsilon_2}}{2}cos(\phi_{b_2}-\phi_{b_1})+\frac{\sqrt{\mu_2\upsilon_2\eta_a\eta_b}}{2}cos(\phi_{b_2}-\phi_{a_2})+\frac{\sqrt{\mu_1\upsilon_2\eta_a\eta_b}}{2}cos(\phi_{b_2}-\phi_{a_1})\\&+\frac{\sqrt{\mu_2\upsilon_1\eta_a\eta_b}}{2}cos(\phi_{b_1}-\phi_{a_2})],\end{aligned}$$

$$
\begin{aligned}
D_{cv}:|B|^2 =&e_d[\frac{\mu_1\eta_a+\mu_2\eta_a}{4}+\frac{\upsilon_1\eta_b+\upsilon_2\eta_b}{4}+\frac{\eta_a\sqrt{\mu_1\mu_2}}{2}cos(\phi_{a_2}-\phi_{a_1})+\frac{\sqrt{\mu_1\upsilon_1\eta_a\eta_b}}{2}cos(\phi_{b_1}-\phi_{a_1})\\
&+\frac{\eta_b\sqrt{\upsilon_1\upsilon_2}}{2}cos(\phi_{b_2}-\phi_{b_1})+\frac{\sqrt{\mu_2\upsilon_2\eta_a\eta_b}}{2}cos(\phi_{b_2}-\phi_{a_2})+\frac{\sqrt{\mu_1\upsilon_2\eta_a\eta_b}}{2}cos(\phi_{b_2}-\phi_{a_1})\\
&+\frac{\sqrt{\mu_2\upsilon_1\eta_a\eta_b}}{2}cos(\phi_{b_1}-\phi_{a_2})],\\
D_{dh}:|C|^2 =&(1-e_d)[\frac{\mu_1\eta_a+\mu_2\eta_a}{4}+\frac{\upsilon_1\eta_b+\upsilon_2\eta_b}{4}+\frac{\eta_a\sqrt{\mu_1\mu_2}}{2}cos(\phi_{a_2}-\phi_{a_1})-\frac{\sqrt{\mu_1\upsilon_1\eta_a\eta_b}}{2}cos(\phi_{b_1}-\phi_{a_1})\\
&+\frac{\eta_b\sqrt{\upsilon_1\upsilon_2}}{2}cos(\phi_{b_2}-\phi_{b_1})-\frac{\sqrt{\mu_2\upsilon_2\eta_a\eta_b}}{2}cos(\phi_{b_2}-\phi_{a_2})-\frac{\sqrt{\mu_1\upsilon_2\eta_a\eta_b}}{2}cos(\phi_{b_2}-\phi_{a_1})\\
&-\frac{\sqrt{\mu_2\upsilon_1\eta_a\eta_b}}{2}cos(\phi_{b_1}-\phi_{a_2})],\\
D_{dv}:|D|^2 =&e_d[\frac{\mu_1\eta_a+\mu_2\eta_a}{4}+\frac{\upsilon_1\eta_b+\upsilon_2\eta_b}{4}+\frac{\eta_a\sqrt{\mu_1\mu_2}}{2}cos(\phi_{a_2}-\phi_{a_1})-\frac{\sqrt{\mu_1\upsilon_1\eta_a\eta_b}}{2}cos(\phi_{b_1}-\phi_{a_1})\\
&+\frac{\eta_b\sqrt{\upsilon_1\upsilon_2}}{2}cos(\phi_{b_2}-\phi_{b_1})-\frac{\sqrt{\mu_2\upsilon_2\eta_a\eta_b}}{2}cos(\phi_{b_2}-\phi_{a_2})\\
&-\frac{\sqrt{\mu_1\upsilon_2\eta_a\eta_b}}{2}cos(\phi_{b_2}-\phi_{a_1})-\frac{\sqrt{\mu_2\upsilon_1\eta_a\eta_b}}{2}cos(\phi_{b_1}-\phi_{a_2})],\\
D_{a_0}:|E|^2 =& \eta_d[\frac{\mu_1}{2}+\frac{\mu_2}{2}-\sqrt{\mu_1\mu_2}cos(\phi_{a_2}-\phi_{a_1})],\quad D_{b_0}:|F|^2=\eta_d[\frac{\upsilon_1}{2}+\frac{\upsilon_2}{2}-\sqrt{\upsilon_1\upsilon_2}cos(\phi_{b_2}-\phi_{b_1})]. \qquad (5)
\end{aligned}
$$

Thus, the detection probability of each threshold SPD is

$$
P_W=1-(1-Y_0)e^{-|W|^2}, \qquad (6)
$$

where $W\in\{A,B,C,D,E,F\}$. To simplify the calculation, we define the following notations:

$$
\begin{aligned}
&y_a=\eta_d(\mu_1+\mu_2),\quad y_b=\eta_d(\upsilon_1+\upsilon_2),\quad x_a=\eta_d\sqrt{\mu_1\mu_2},\quad x_b=\eta_d\sqrt{\upsilon_1\upsilon_2},\quad y_1=\eta_a(\mu_1+\mu_2),\\
&y_2=\eta_b(\upsilon_1+\upsilon_2),\quad x_1=\eta_a\sqrt{\mu_1\mu_2},\quad x_2=\eta_b\sqrt{\upsilon_1\upsilon_2},\quad x_{11}=\sqrt{\eta_a\eta_b\mu_1\upsilon_1},\quad x_{12}=\sqrt{\eta_a\eta_b\mu_1\upsilon_2},\\
&x_{21}=\sqrt{\eta_a\eta_b\mu_2\upsilon_1},\quad x_{22}=\sqrt{\eta_a\eta_b\mu_2\upsilon_2},\quad \phi_a=\phi_{a_2}-\phi_{a_1},\quad \phi_b=\phi_{b_2}-\phi_{a_2},\quad \phi_{ab}=\phi_{b_1}-\phi_{a_1}.
\end{aligned} \qquad (7)
$$

Then, the detection probability of each threshold SPD can be expressed by

$$
\begin{aligned}
P_A =&1-(1-Y_0)exp\{-(1-e_d)\{\frac{1}{4}(y_1+y_2)+\frac{1}{2}[x_1cos\phi_a+x_2cos\phi_b+x_{11}cos\phi_{ab}\\
&+x_{22}cos(\phi_b-\phi_a+\phi_{ab})+x_{12}cos(\phi_{ab}+\phi_b)+x_{21}cos(\phi_{ab}-\phi_a)]\}\},\\
P_B =&1-(1-Y_0)exp\{-e_d\{\frac{1}{4}(y_1+y_2)+\frac{1}{2}[x_1cos\phi_a+x_2cos\phi_b+x_{11}cos\phi_{ab}\\
&+x_{22}cos(\phi_b-\phi_a+\phi_{ab})+x_{12}cos(\phi_{ab}+\phi_b)+x_{21}cos(\phi_{ab}-\phi_a)]\}\},\\
P_C =&1-(1-Y_0)exp\{-(1-e_d)\{\frac{1}{4}(y_1+y_2)+\frac{1}{2}[x_1cos\phi_a+x_2cos\phi_b-x_{11}cos\phi_{ab}\\
&-x_{22}cos(\phi_b-\phi_a+\phi_{ab})-x_{12}cos(\phi_{ab}+\phi_b)-x_{21}cos(\phi_{ab}-\phi_a)]\}\},\\
P_D =&1-(1-Y_0)exp\{-e_d\{\frac{1}{4}(y_1+y_2)+\frac{1}{2}[x_1cos\phi_a+x_2cos\phi_b-x_{11}cos\phi_{ab}\\
&-x_{22}cos(\phi_b-\phi_a+\phi_{ab})-x_{12}cos(\phi_{ab}+\phi_b)-x_{21}cos(\phi_{ab}-\phi_a)]\}\},\\
P_E =& 1-(1-Y_0)exp(-\frac{1}{2}y_a+x_acos\phi_a),\quad P_F=1-(1-Y_0)exp(-\frac{1}{2}y_b+x_bcos\phi_b). \qquad (8)
\end{aligned}
$$

To further simplify the expressions of detection probability, we give some new notations:

$$h_a=\frac{1}{2}y_a-x_a cos\phi_a,\quad h_b=\frac{1}{2}y_b-x_b cos\phi_b, g=\frac{1}{4}(y_1+y_2)+\frac{1}{2}(x_1 cos\phi_a+x_2 cos\phi_b),$$
$$h=\frac{1}{2}[x_{11}cos\phi_{ab}+x_{22}cos(\phi_b-\phi_a+\phi_{ab})+x_{12}cos(\phi_{ab}+\phi_b)+x_{21}cos(\phi_{ab}-\phi_a)]. \tag{9}$$

Then, we have

$$P_A=1-(1-Y_0)e^{-(1-e_d)(g+h)},\quad P_B=1-(1-Y_0)e^{-e_d(g+h)},\quad P_C=1-(1-Y_0)e^{-(1-e_d)(g-h)},$$
$$P_D=1-(1-Y_0)e^{-e_d(g-h)},\quad P_E=1-(1-Y_0)e^{-h_a},\quad P_F=1-(1-Y_0)e^{-h_b}. \tag{10}$$

We can derive that

$$Q^{\lambda}_{HH,\psi^+}=2P_AP_B(1-P_C)(1-P_D)T(i,j),\quad Q^{\lambda}_{HH,\psi^-}=2P_AP_D(1-P_B)(1-P_c)T(i,j),$$
$$T(i,j)=P_E^i(1-P_E)^{1-i}P_F^j(1-P_F)^{1-j}, \tag{11}$$

where Q^{λ}_{HH,ψ^+} and Q^{λ}_{HH,ψ^-} denote, respectively, the probability of the projection on the $|\psi^+\rangle=\frac{1}{\sqrt{2}}(|H,V\rangle+|V,H\rangle)$ and the $|\psi^-\rangle=\frac{1}{\sqrt{2}}(|H,V\rangle-|V,H\rangle)$. Here from figure 1, $|\psi^+\rangle$ means the coincident detections of $\{ch$ & $cv\}$ or $\{dh$ & $dv\}$; $|\psi^-\rangle$ means the coincident detections of $\{ch$ & $dv\}$ or $\{cv$ & $dh\}$. $T(i,j)$ is a function of i and j expressing the probability that Alice prepares a c_i-mode state while Bob prepares a c_j-mode state; it depends on the states (signal state or decoy state) Alice and Bob choose. Thus, we can obtain Q^{λ}_{HH} by

$$Q^{\lambda}_{HH}=Q^{\lambda}_{HH,\psi^+}+Q^{\lambda}_{HH,\psi^-}. \tag{12}$$

It has the form

$$Q^{\lambda}_{HH}=2T(i,j)[2(1-Y_0)^4e^{-2g}-(1-Y_0)^3e^{e_dg+e_dh-2g}-2(1-Y_0)^3e^{-e_dg-e_dh-g+h}$$
$$-(1-Y_0)^3e^{e_dg-e_dh-2g}+(1-Y_0)^2e^{h-g}+(1-Y_0)^2e^{h-g-2e_dh}]. \tag{13}$$

In the similar way, we can obtain Q^{λ}_{HV} expressed by

$$Q^{\lambda}_{HV}=2T(i,j)[2(1-Y_0)^4e^{-2g}-2(1-Y_0)^3e^{-2g_1+e_dg-g_2-e_dg_2-\sqrt{e_d(1-e_d)}h}$$
$$-(1-Y_0)^3e^{-g_1-e_dg_1-2g_2+e_dg_2-\sqrt{e_d(1-e_d)}h}-(1-Y_0)^3e^{-g_1-e_dg_1-2g_2+e_dg_2+\sqrt{e_d(1-e_d)}h}$$
$$+(1-Y_0)^2e^{-g+2\sqrt{e_d(1-e_d)}h}+(1-Y_0)^2e^{-g}], \tag{14}$$

where $g_1=\frac{1}{4}y_1+\frac{1}{2}x_1cos\phi_a$ and $g_2=\frac{1}{4}y_2+\frac{1}{2}x_2cos\phi_b$.

We know that total gain $Q^{\lambda}_{c_ic_j}$ is independent of ϕ_a, ϕ_b, and ϕ_ab, so we can take the integrals of ϕ_a, ϕ_b, and ϕ_ab for the expression of $Q^{\lambda}_{c_ic_j}$:

$$Q^{\lambda}_{c_ic_j}=\frac{1}{8\pi^3}\int_0^{2\pi}\int_0^{2\pi}\int_0^{2\pi}\frac{Q^{\lambda}_{HH}+Q^{\lambda}_{HV}}{2}d\phi_ad\phi_bd\phi_{ab}. \tag{15}$$

We can get the error rate $E^{\lambda}_{c_ic_j}$ by

$$E^{\lambda}_{c_ic_j}=\frac{1}{8\pi^3}\int_0^{2\pi}\int_0^{2\pi}\int_0^{2\pi}\frac{Q^{\lambda}_{HH}}{Q^{\lambda}_{HH}+Q^{\lambda}_{HV}}d\phi_ad\phi_bd\phi_{ab}. \tag{16}$$
